# Exacerbations and Changes in Physical Activity and Sedentary Behaviour in Patients with Bronchiectasis after 1 Year

**DOI:** 10.3390/jcm10061190

**Published:** 2021-03-12

**Authors:** Victoria Alcaraz-Serrano, Ane Arbillaga-Etxarri, Patricia Oscanoa, Laia Fernández-Barat, Leticia Bueno, Rosanel Amaro, Elena Gimeno-Santos, Antoni Torres

**Affiliations:** 1 Fundació Clínic per la Recerca Biomèdica (FCRB), CIBERES, Hospital Clínic de Barcelona, Villarroel Street 170, 08036 Barcelona, Spain; victoriaalcarazserrano@gmail.com (V.A.-S.); lfernan1@clinic.cat (L.F.-B.); bueno@clinic.cat (L.B.); atorres@clinic.cat (A.T.); 2 Physiotherapy Department, University of Deusto, Mundaiz Street 50, 20012 Donostia San Sebastián, Gipuzkoa, Spain; ane.arbillaga@deusto.es; 3 Institut Clínic Respiratori, Hospital Clínic de Barcelona, Villarroel Street 170, 08036 Barcelona, Spain; oscanoa@clinic.cat (P.O.); ramaro@clinic.cat (R.A.); 4 Institut d’Investigacions Biomèdiques August Pi i Sunyer (IDIBAPS), Hospital Clinic de Barcelona, Villarroel Street 170, 08036 Barcelona, Spain; 5 Non-Communicable Diseases and Environment Department, Barcelona Institute for Global Health (ISGlobal). Dr. Aiguader Street 88, 08003 Barcelona, Spain

**Keywords:** bronchiectasis, physical activity, sedentary behaviour, exacerbation

## Abstract

Background: Low physical activity and high sedentary behaviour in patients with bronchiectasis are associated with hospitalisation over one year. However, the factors associated with longitudinal changes in physical activity and sedentary behaviour have not been explored. We aimed to identify clinical and sociodemographic characteristics related to a change in physical activity and sedentary behaviour in patients with bronchiectasis after one year. Methods: This was a prospective observational study during which physical activity measurements were recorded using a SenseWear Armband for one week at baseline and at one year. At each assessment point, patients were classified as active or inactive (measured as steps per day) and as sedentary or not sedentary (measured as sedentary time). Results: 53 patients with bronchiectasis were analysed, and after one year, 18 (34%) had worse activity and sedentary levels. Specifically, 10 patients became inactive and sedentary. Multivariable analysis showed that the number of exacerbations during the follow-up period was the only outcome independently associated with change to higher inactivity and sedentary behaviour (odds ratio (OR), 2.19; 95% CI, 1.12 to 4.28). Conclusions: The number of exacerbations in patients with bronchiectasis was associated with changes in physical activity and sedentary behaviour. Exacerbation prevention may appear as a key factor in relation to physical activity and sedentary behaviour in patients with bronchiectasis.

## 1. Introduction

Bronchiectasis is a chronic respiratory disease characterised by chronic productive cough, dyspnoea, and frequent exacerbations [1]. An exacerbation is defined as a person with bronchiectasis with a deterioration in three or more of the following key symptoms for at least 48 h: cough, sputum volume and/or consistency, sputum purulence, breathlessness and/or exercise tolerance, fatigue and/or malaise, haemoptysis; and a clinician determines that a change in treatment is required [2]. Exacerbations increase the severity of microbiological, radiological, and functional outcomes [3], being differentiated into mild, when patients are treated with oral antibiotics as outpatients, and moderate to severe, when they require hospitalisation and intravenous therapy [4]. Furthermore, a higher frequency of exacerbations is associated with an increased risk of mortality [5].

It is known that in patients with chronic obstructive pulmonary disease (COPD), high levels of physical activity and low time in sedentary behaviour are at a reduced risk of exacerbations and reduced health care utilisation, which leads to various cost savings [6,7]. By contrast, there is limited knowledge of the relation between physical activity, sedentary behaviour, and exacerbations in patients with bronchiectasis. It was recently described that patients with bronchiectasis who spent ≥7.8 h/day in sedentary behaviour were at a 5.9 times greater risk of future severe exacerbations [8]. Physical activity is a complex behaviour structured in variables according to different intensities and outcomes [9]. Although steps per day and time spent in sedentary behaviour are strongly correlated, they should be considered independently because they each have their own peculiarities and associated factors [10]. To date, the factors associated with the modification in physical activity and sedentary behaviour after one year have not been explored for either measure in patients with bronchiectasis.

Therefore, the aim of this study was to analyse the clinical and sociodemographic characteristics associated with a change in physical activity and sedentary behaviour after one year in patients with bronchiectasis.

## 2. Materials and Methods

### 2.1. Study Design

This was a prospective observational study conducted at the pulmonology service of a tertiary care hospital in Barcelona, Spain. Physical activity and sedentary behaviour measurements were performed at baseline and at one year follow-up to study the distribution of groups (active/inactive and sedentary/not sedentary) and their longitudinal modification. We also investigated the clinical and sociodemographic factors potentially related to a shift between physical activity and sedentary behaviour levels. The inclusion criteria were as follows: (1) adults (≥18 years of age) diagnosed with bronchiectasis, as confirmed by computed tomography and with symptoms of the disease; (2) clinical stability (no exacerbations and no significant change in symptoms and/or medication in the last four weeks); (3) the ability to perform all the clinical tests and understand the process and the purposes of the study; (4) willing to give informed consent. Exclusion criteria were: (1) any physical or psychological disorder that might interfere with protocol compliance; (2) diagnosis of cystic fibrosis, sarcoidosis, pulmonary fibrosis, active tuberculosis (TB), or non-TB mycobacterial infection in treatment; (3) participation in a pulmonary rehabilitation (PR) programme in the last year; (4) respiratory insufficiency and/or oxygen therapy; (5) missing data after 12 months of follow-up.

The study was approved by the Clinical Research Ethics Committee of the Hospital Clinic (Ethics Approval Reference: HCB/2016/0012). Informed consent was obtained from all subjects involved in the study.

### 2.2. Measurements

Patients were assessed at baseline and after one year, with no intervention provided during this year. Dyspnoea was measured using the modified Medical Research Council (mMRC) scale [11]. Lung function was assessed with an EasyOne^TM^ World Spirometer (NDD Medical Technologies, Zurich, Switzerland) and classified according to the American Thoracic Society/European Respiratory Society Guidelines [12]. Exercise capacity was measured using the 6-min walking test (6MWT) [13]. Quality of life was assessed using the Quality-of-Life Bronchiectasis questionnaire (QoL-B) [14], while the impact of coughing on the quality of life was assessed with the Leicester Cough Questionnaire (LCQ) [15]. Bronchiectasis severity was assessed using the Bronchiectasis Severity Index (BSI) Score [16]. We recorded the number of exacerbations and hospitalisations in the medical dataset during the follow-up based on a consensus definition [2]. Frequency of exacerbations for sub-analysis was divided into 0, 1–2, and ≥3 exacerbations during the follow-up [17].

Physical activity and sedentary behaviour were measured using a tri-axial accelerometer, the SenseWear Armband (SWA) (BodyMedia Inc., Pittsburgh, PA, USA). Participants were asked to wear the SWA for the maximum time possible over seven days, except during water-based activities. It was worn in the triceps area, on the rear of the dominant arm [18]. Intensity of physical activity was reported as metabolic equivalents (METs) and classified into sedentary (≤1.5 MET), light (1.6 to <3.0 MET), moderate (3.0 to <6.0 MET), and vigorous (≥6.0 MET) [19]. The mean time (in minutes) spent at each intensity level was recorded. Moderate-to-vigorous physical activity (MVPA) was calculated as the mean number of minutes spent in moderate and vigorous physical activity on the valid days. Sedentary time was analysed as the number of minutes the patient spent at ≤1.5 MET intensity.

Patients were classified into four groups according to their physical activity and sedentary behaviour levels at baseline and at one year. The cut-off for being active was ≥6290 steps per day and for being sedentary ≥7.8 h per day spent in sedentary behaviour [8]. The groups were labelled from the best to worst, as follows: ‘active + not sedentary’, ‘active + sedentary’, ‘inactive + not sedentary’, ‘inactive + sedentary’. The dependent variable was the change, after one year follow-up, to the worst group ‘inactive + sedentary’.

### 2.3. Statistical Analysis

Data are presented as numbers (%) for categorical variables, as means ± standard deviations (SD) for normally distributed data and as medians (P_25_–P_75_) (1st and 3rd quartiles) for non-normally distributed data. The assumption of normality was checked by means of Shapiro–Wilk tests. Comparisons of categorical variables were performed using the chi-square test. Comparisons between continuous variables were performed by analysis of variance (ANOVA) or the Kruskal–Wallis test. If the overall ANOVA (or Kruskal–Wallis) result was significant, we conducted post-hoc pairwise comparisons with Bonferroni correction to control for the experiment-wise error rate.

Logistic regression analyses [20] were used to examine the associations between the change to ‘inactive + sedentary’ and the various risk factors. In the first step, each risk factor, together with the numbers of hospitalisations and exacerbations during the follow-up, were tested individually. We included age, 6-min walking distance, and forced expiratory volume in the first second predicted (FEV_1_%) as risk factors. In the second step, all risk factors that showed an association in the univariate model (*p* < 0.10) were added to the multivariable model. Finally, a backward stepwise selection (likelihood ratio) (pin < 0.05, pout > 0.10) was used to determine factors associated with change to the ‘inactive + sedentary’ group after one year [21]. Multicollinearity was assessed by calculating the variance inflation factor, and we calculated the odds ratios (ORs) and their 95% confidence intervals (CIs). The Hosmer–Lemeshow goodness-of-fit test was performed to assess the overall fit of the final model. The internal validity of the final model was assessed using ordinary non-parametric bootstrapping with 1000 bootstrap samples and bias-corrected, accelerated 95% CIs. 

The level of significance was set at 0.05 (two-tailed) for all analyses, which were performed using IBM SPSS Statistics 26.0 (IBM Corp., Armonk, NY, USA).

## 3. Results

### 3.1. Baseline Data

**Figure 1:**
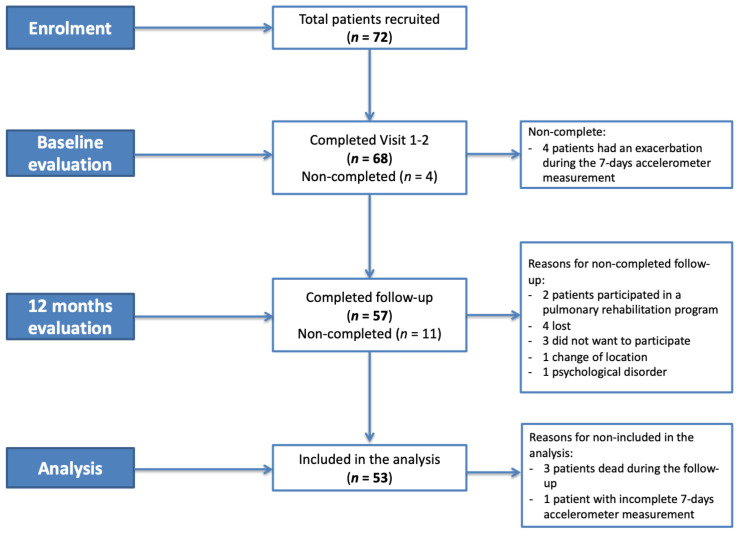
Enrolment flow-chart.

Of the 72 patients with bronchiectasis who we recruited, 53 (37 females, mean age 62 ± 16 years) were included in the analysis (Figure 1).

Their baseline sociodemographic and clinical characteristics are shown in Table 1, with 24 in the ‘active + not sedentary’ group, 3 in the ‘active + sedentary’ group, 11 in the ‘inactive + not sedentary’ group, and 15 in the ‘inactive + sedentary’ group. There were only differences in physical activity outcomes where the group ‘inactive + sedentary’ had the lowest values of light and moderate physical activity, MVPA and steps per day, as well as the highest value for sedentary time.

### 3.2. Follow-Up Assessment at 1 Year

After one year follow-up, the number of patients in each group changed to 18 in the ‘active + not sedentary’ group, 7 in the ‘active + sedentary’ group, 8 in the ‘inactive + not sedentary’ group, and 20 in the ‘inactive + sedentary’ group. In total, 27 (51%) patients changed to a different group, of whom only 9 (17%) improved from a sedentary and/or inactive group to a not sedentary and/or active group. The other 18 (34%) patients deteriorated, and 10 of these shifted to the worst group (‘inactive + sedentary’). At this time, the ‘inactive + sedentary’ group had more exacerbations, lower physical activity levels, and worse quality of life (Table 2).

### 3.3. Factors Associated with the Shift to Reduced Activity Levels

Results from the multivariable analysis showed that the number of exacerbations during the follow-up (OR, 2.19; 95% CI, 1.12 to 4.28) was independently associated with change to the ‘inactive + sedentary’ group (Table 3). Internal validation of the logistic regression model using bootstrapping with 1000 samples demonstrated robust results for the variable included in the model, with small 95% CIs around the original coefficients.

### 3.4. Relationship between Baseline and Follow-Up Activity Levels by Number of Exacerbations

**Figure 2:**
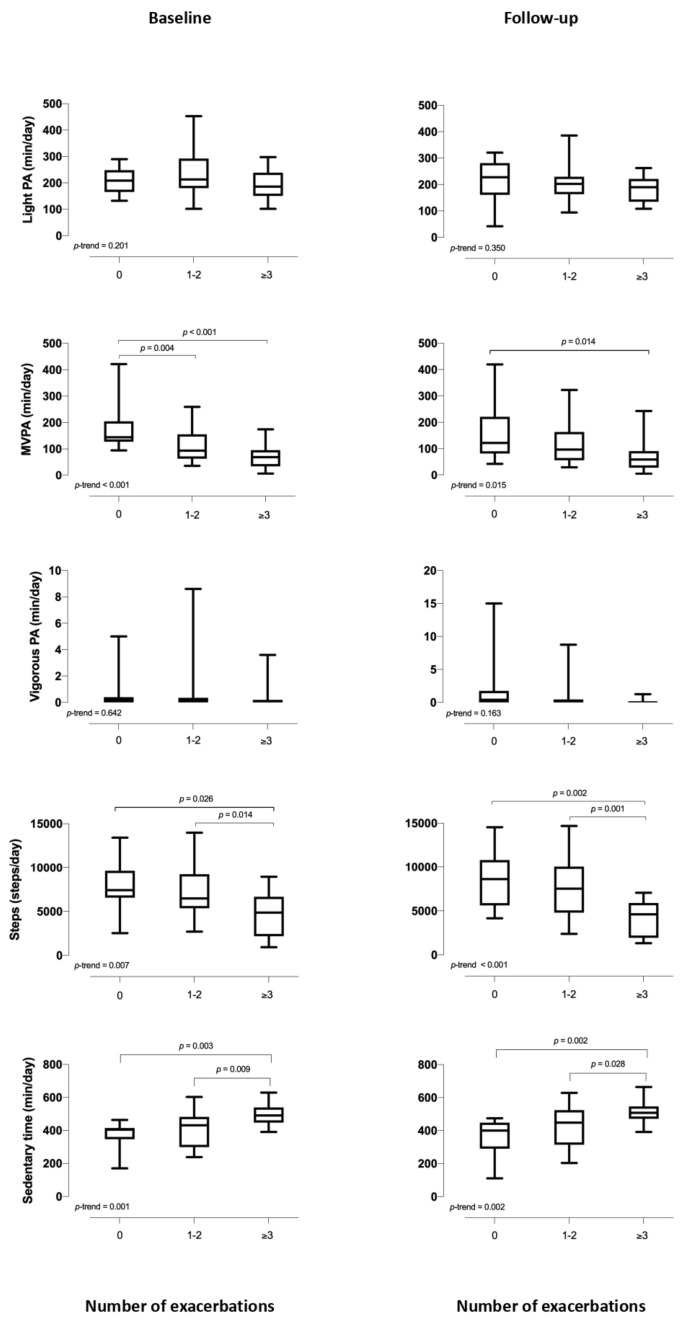
Physical activity and sedentary behaviour at baseline and follow-up according to number of exacerbations. The box and whisker plots show the mean ± SD for each physical activity variable and sedentary time by the number of exacerbations (0, 1–2, and ≥3). The left and right figures show the data for baseline and one year, respectively. Abbreviations: min, minutes; MVPA, moderate-to-vigorous physical activity; PA, physical activity.

In the final cohort, 11 (21%) patients had 0 exacerbations, 25 (47%) had 1–2 exacerbations, and 17 (32%) had ≥3 exacerbations. The physical activity and sedentary behaviour variables at baseline and at one year, together with the ***p***-trend for each parameter, are shown in Figure 2. At both evaluations, the group with ≥3 exacerbations had the lowest MVPA, lowest number of steps per day and the highest sedentary time compared with the other two groups (i.e., 0 or 1–2 exacerbations).

## 4. Discussion

To the best of our knowledge, this is the first study to have (1) evaluated and categorised patients with bronchiectasis by their physical activity and sedentary levels at baseline and one year, (2) identified and characterised those patients who deteriorated to become most ‘inactive + sedentary’ after one year, and (3) associated this shift to the number of exacerbations during the follow-up.

In our previous study, patients with bronchiectasis who walked ≤6290 steps day and/or spent ≥7.8 h in sedentary behaviour were at higher risk of hospitalisation in the following year; concluding that physical activity and sedentary behaviour were a determinant for hospitalisation of bronchiectasis [8]. In the present study, the number of exacerbations during the follow-up was a determinant for changing physical activity and sedentary behaviour in patients with bronchiectasis. Likewise, Bradley et al. [22] reported that patients with bronchiectasis and low physical activity levels showed increased severity, which may have been related to their physical impairment, thereby more exacerbations. Martínez-García et al. [5] also showed that there is a specific group of patients with bronchiectasis who are characterised by a high frequency of exacerbations (at least two) or a hospitalisation per year; specifically, it was shown that these patients had a worse five-year all-cause mortality independent to the initial severity of bronchiectasis. These data, combined with the present results, indicate that a high frequency of exacerbations may be associated with declining physical activity levels and increasing sedentary behaviour over time rather than with the severity of exacerbations (reflected by hospitalisation).

In similar populations, such as COPD, Moy et al. [23] showed that lower daily step count was associated with significantly higher rates of acute exacerbations and hospitalisations. Donaire et al. [24] also reported that an increase of 1000 steps daily, performed at low average intensity, reduced the risk of hospitalisation by 20%, whereas Nguyen et al. [25] reported that there was a significant risk reduction (34%) in 30-day readmissions among patients with COPD who reported engaging in any physical activity. Regarding sedentary behaviour, this has been associated with more exacerbations [26] and higher mortality [27]. Previous observational studies have consistently shown that physical inactivity is associated with an increased risk of hospitalisations and mortality in patients with COPD [28,29,30,31,32]. According to our results, the pattern of decline in physical activity and increase in sedentary behaviour was associated with the number of exacerbations in bronchiectasis; hence, we hypothesise that this may be strongly associated with a worse prognosis. Therefore, these findings highlight the necessity of including assessments for both physical activity and sedentary behaviour in routine clinical practice. PR programs have demonstrated improvements in exercise capacity and health related quality of life in patients with bronchiectasis. However, there is no evidence related to the sustainability of the effects achieved by the exercise training and the importance of the behaviour change as a challenge in the management of patients with bronchiectasis [33]. Hence, more observational and interventional studies are also needed to describe and improve physical activity and sedentary behaviour in the bronchiectasis population. In terms of physical activity intensity, the group with ≥3 exacerbations were least active (lowest MVPA and steps per day, with the highest sedentary time) compared with the groups that had 0 or 1–2 exacerbations during follow-up. There was no difference when assessing light physical activity and exercise capacity. This finding is consistent with our previous study where hospitalised patients walked fewer steps per day, spent more time being sedentary, and had lower levels of MVPA compared with their peers who were not hospitalised. Although the intensity of physical activity seems related to a reduction in exacerbation risk and disease severity, more studies are needed for clarification. Bradley et al. [21] reported that MVPA in bouts of ≥10min correlated with QoL-B Social Functioning and that patients with moderate/severe disease spent significantly less time in daily total MVPA. In our study, social, physical, and role function domains of the QoL-B were statistically different between groups at follow-up, and these differences were greater than the minimal important difference [34]. It seems that low physical activity and high sedentary behaviour could be associated with a higher decline in quality of life after one year of follow-up.

Exacerbations, with or without hospitalisation, hasten acute physical activity deterioration and inactivity in patients with COPD [35,36,37,38,39,40]. However, the determinants of physical activity change over time are poorly understood [5]. In our longitudinal analysis, 18 patients (34%) switched from more active or non-sedentary groups to more inactive and/or sedentary groups over one year. Among those patients, 10 (55%) switched directly to the worst group, and except for the number of exacerbations, not other clinical or functional characteristic (i.e., BSI score, lung function, dyspnoea, or exercise capacity) was associated with this change. Likewise, Bradley et al. [21] reported that neither lung function (FEV_1_% predicted) nor disease severity (BSI score) was correlated with sedentary behaviour in patients with bronchiectasis. Data collected from patients with COPD have shown that physical activity decliners vary from 35% to 59% over time [41,42], but the clinical characteristics could not predict or explain the subsequent patterns of decline, included the number of exacerbations [42,43,44,45]. This indicates that the common clinical and functional assessments are unsuitable for use as indicators of risk of physical activity decline and/or increase in sedentary behaviour, which further highlights the need for further longitudinal and objective assessment in clinical settings. Moving forward, the whole spectrum of those behaviours must be included, accounting for the potential impacts of psychological, interpersonal, social, and environmental correlates in the assessment [5,42,46].

A major strength of our study is the use of validated and objective devices to assess physical activity and sedentary behaviour over a one year follow-up period. The results provide novel and valuable information that will require allied respiratory health professionals to design interventions that can enhance physical activity and reduce sedentary behaviour in patients with bronchiectasis.

However, the study also has limitations. The sample size (*n* = 53) may result in a large type II error and conclusions that can be drawn are limited. This limitation notwithstanding, the rigorous approach to the study underpins our confidence in its findings and their clinical relevance. In addition, possible over-fitting and instability of the variables due to the limited sample size in the logistic regression model evaluating the change in physical activity and sedentary behaviour after one year in patients with bronchiectasis, was measured by internal validation using ordinary nonparametric bootstrapping, which demonstrated robust results. Other measurements related to physical activity and sedentary behaviour, such as muscle strength, were missing and may have improved the analysis. This should be addressed in future research. Considering the heterogeneity of physical activity and sedentary behaviour levels among countries and cultures, the study results may not be generalizable to other settings. Further studies are, therefore, needed, with larger number of patients included that target different bronchiectasis populations in other regions or medical settings.

## 5. Conclusions

In this broad assessment of clinical, functional, and sociodemographic factors, a decline in physical activity levels and increase in sedentary behaviour over one year in patients with bronchiectasis was independently associated with the number of exacerbations. Exacerbation prevention may appear as a key factor in relation to physical activity and sedentary behaviour in patients with bronchiectasis.

## Figures and Tables

**Table 1:** Baseline characteristics by physical activity and sedentary behaviour classification.

	All Patients	‘Active + Not Sedentary’	‘Active + Sedentary’	‘Inactive + Not Sedentary’	‘Inactive + Sedentary’	*p* Value
	N = 53	24 (45)	3 (6)	11 (21)	15 (28)	
**Demographics**						
Female	37 (70)	19 (79)	1 (33.3)	7 (63.6)	10 (66.6)	0.368
Age, years	62.3 (15.9)	58.7 (14.9)	62.7 (6.4)	64.6 (21.9)	66.1 (13.9)	0.523
BMI, Kg/m^2^	24.3 (4.1)	23.8 (3.9)	29.4 (5.1)	23.9 (2.9)	24.1 (4.6)	0.174
**Work activity**						0.263
Active	19 (36)	12 (50)	1 (33.3)	2 (18.2)	4 (26.7)	
Retired	34 (64)	12 (50)	2 (66.6)	9 (81.8)	11 (73.3)	
**Smoking habit**						0.538
Active smoker	1 (2)	1 (4.2)	0 (0)	0 (0)	0 (0)	
Former smokers	14 (26)	4 (16.6)	2 (66.6)	3 (27.2)	5 (33.3)	
Non-smoker	38 (71.7)	19 (79.2)	1 (33.3)	8 (72.7)	10 (66.6)	
Chronic colonisation	20 (37)	5 (20.8)	1 (33.3)	6 (51.5)	8 (53.3)	0.121
*Pseudomonas aeruginosa*	16 (30)	3 (60)	1 (100)	5 (83.3)	7 (87.5)	0.081
Dyspnoea (mMRC Scale, 0–4)	1 [1,1]	1 [0,1]	1 [1,1]	1 [1,2]	1 [1,2]	0.263
No. exacerbations previous year	1 [0,1,2]	1 [0,1,2]	1 [0,1]	2 [0,1,2]	1 [0,1,2,3,4]	0.283
No. hospitalizations previous year	0 [0,0]	0 [0,0]	0 [0,0]	0 [0,0]	0 [0,1]	0.653
Number of lobes affected in CT scan	3.62 (1.6)	3.50 (1.7)	2.33 (0.6)	3.91 (1.3)	3.86 (1.7)	0.350
**Aetiology**						
Post-infectious	24 (45)	15 (62.5)	0 (0)	6 (54.5)	3 (20)	0.022
Idiopathic	13 (24)	3 (12.5)	2 (66.6)	2 (18)	6 (40)	0.074
Others	16 (30.2)	6 (25)	1 (33.3)	3 (27.3)	6 (40)	0.789
**Severity**						
BSI stages	2 [1,2,3]	2 [1,2]	2 [1,2]	2 [2,3]	3 [1,2,3]	0.086
**Pulmonary function**						
FEV1, % predicted	73.2 (20)	80.9 (18.2)	67 (5.56)	68.9 (23.1)	65.4 (19.9)	0.086
FVC, % predicted	81 (18.3)	87.2 (16.4)	90.3 (38.6)	72.36 (14.3)	75.6 (16.5)	0.059
FEV1/FVC, %	85.9 (18)	89.1 (17.2)	73 (17.7)	84.9 (19.4)	84.2 (18.6)	0.490
**6MWT, metres**	516.8 (97.8)	534.3 (80.03)	564.8 (40.5)	512.8 (103.4)	482.3 (122.01)	0.345
**Physical activity**						
Light (min per day)	216.7 (77)	237.2 (71.6)	138.8 (64.4)	256.6 (91.9)	170.2 (37.6)	0.002 ^a,b^
Moderate (min per day)	112 (76)	143.3 (91.1)	97.2 (24.5)	117.5 (57.4)	60.8 (30.1)	0.008 ^a^
Vigorous (min per day)	0.66 (1.7)	1.1 (2.3)	0 (0)	0.26 (0.31)	0.44 (1.0)	0.445
MVPA (min per day)	112.5 (77)	144 (92.1)	97.2 (24.5)	117.7 (57.5)	61.2 (30.5)	0.009 ^a^
Steps per day	6759 (3530)	9441 (3014)	8912 (549)	4840 (1120)	3444 (1563)	<0.001
Sedentary time (min)	430 (99.8)	374.4 (75.7)	487.5 (18.4)	384.9 (79.8)	541 (46.6)	<0.001 ^a,b^
**Quality of Life Bronchiectasis Questionnaire**						
Physical Function	60.4 (32.7)	62.3 (33.3)	55.5 (19.2)	45.5 (34.2)	64.4 (32)	0.382
Role Function	76.7 (26.6)	83.3 (26)	66.6 (0)	75.7 (26.2)	68.9 (29.5)	0.367
Vitality	59.4 (26.8)	62.5 (24.7)	55.6 (19.2)	65.2 (24)	51.1 (33)	0.521
Emotional Function	74.2 (28.9)	75.7 (27.4)	61.1 (25.4)	69.7 (34)	77.8 (30)	0.771
Social Function	69.8 (30.8)	76.4 (24.5)	55.6 (38.5)	65.2 (36.9)	65.6 (34.7)	0.530
Treatment Burden	72.9 (39.8)	72.2 (42.5)	66.7 (57.7)	69.7 (37.8)	77.7 (37)	0.948
Health Perceptions	53.1 (26.7)	61.8 (28.4)	50 (16.7)	51.5 (24.1)	41.4 (24.3)	0.129
Respiratory Symptoms	76.7 (23.2)	77.7 (23.4)	66.7 (33.3)	78.8 (22.5)	75.6 (23.5)	0.981
**Leicester Cough Questionnaire**						
Total	15.3 (4.64)	16.04 (4.2)	12.9 (7.2)	15.1 (5.5)	14.8 (4.5)	0.678
Physical	4.9 (1.34)	5.2 (1.2)	4.37 (2.3)	5.03 (1.3)	4.74 (1.5)	0.616
Psychological	5.01 (1.74)	5.2 (1.6)	4.19 (2.4)	4.78 (2.2)	5.04 (1.6)	0.773
Social	5.44 (1.8)	5.67 (1.6)	4.41 (2.7)	5.3 (2.2)	5.4 (1.6)	0.710

Abbreviations: 6MWT, 6-min walking test; BMI, body mass index; BSI, bronchiectasis severity index; CT: computed tomography; FEV_1_, forced expiratory volume in 1 s; FVC, forced vital capacity; Kg, kilogram; m, metre; min: minutes; mMRC, modified Medical Research Council scale; MVPA, moderate-to-vigorous physical activity. Data are presented as n (%), mean ± SD or median (P_25_–P_75_). ^a^ *p* < 0.05 for comparison between ‘active + not sedentary’ vs. ‘inactive + sedentary’. ^b^ *p* < 0.05 for comparison between ‘inactive + sedentary’ vs. ‘inactive + not sedentary’.

**Table 2:** Follow-up characteristics by physical activity and sedentary behaviour classification.

	All Patients	‘Active + Not Sedentary’	‘Active + Sedentary’	‘Inactive + Not Sedentary’	‘Inactive + Sedentary’	*p* Value
	N = 53	18 (34)	7 (13)	8 (15)	20 (38)	
**Demographics**						
BMI, Kg/m^2^	24.2 (4.3)	25.1 (4.2)	24.2 (4.6)	21.6 (4.1)	24.7 (4.4)	0.287
Dyspnoea (mMRC Scale, 0–4)	1 [0,1]	1 [0,1,2]	1 [0.75–2.25]	2 [0,1,2,3]	3 [2,3,4]	0.075
No. exacerbations during FUP	2 [1,2,3]	1 [0,1,2]	1 [0.75–2.25]	2 [0,1,2,3]	3 [2,3,4]	<0.001
No. hospitalisations during FUP	0 [0–0.5]	0 [0,0]	0 [0,0]	0 [0–0.75]	0 [0,1]	0.125
**Pulmonary function**						
FEV_1_, % predicted	69.5 (20.4)	75.5 (19.4)	73.8 (22.4)	64.1 (23.2)	66.7 (19.1)	0.363
FVC, % predicted	77.7 (17.8)	82.4 (16.6)	77.4 (13.5)	72.7 (23.1)	76.5 (18.2)	0.256
FEV_1_/FVC, %	83.7 (18.3)	83.9 (14.8)	93 (20.5)	87.4 (30.7)	81.9 (13.3)	0.532
**6MWT, metres**	520.4 (98.5)	540.3 (77.2)	582.9 (81.2)	505.4 (73.9)	470.8 (111.8)	0.068
**Physical activity**						
Light (min per day)	205.4 (75.5)	248.2 (86.1)	164.8 (37.1)	206.1 (86.4)	179.2 (53.7)	0.016 ^a^
Moderate (min per day)	107.6 (84.2)	160.2 (95.7)	86.2 (36.3)	155.7 (88.8)	49.8 (24.6)	<0.001 ^a,c^
Vigorous (min per day)	0.89 (2.51)	1.9 (3.9)	1.14 (2.3)	0.5 (0.67)	0.04 (0.14)	0.123
MVPA (min per day)	108.5 (85.1)	162.2 (96.6)	87.3 (37.4)	156.2 (89.4)	49.9 (24.6)	<0.001
Steps per day	6781 (5799)	10443 (2681)	8073 (1624)	4383 (1764)	3983 (1484)	<0.001 ^a,c^
Sedentary time (min)	441.2 (115.6)	337.9 (72.1)	501.3 (30.3)	372.6 (117.1)	540.2 (53.3)	<0.001
						
**Quality of Life Bronchiectasis Questionnaire**						
Physical Function	53.14 (31.8)	61.1 (23.6)	72.2 (25.1)	50 (34.7)	35.1 (32.3)	0.026
Role Function	72.01 (29.56)	81.5 (23.5)	94.4 (13.6)	61.9 (12.6)	57 (35.7)	0.012 ^a,b^
Vitality	59.4 (22.98)	61.1 (21.4)	63.8 (16.4)	66.7 (21.5)	50.8 (25.7)	0.215
Emotional Function	74.5 (27.5)	73.1 (24.3)	88.9 (17.2)	80.9 (26.2)	66.7 (33.3)	0.295
Social Function	64.5 (31.4)	78.7 (23.4)	75 (20.4)	54.7 (20.9)	48.2 (37.6)	0.030 ^a^
Treatment Burden	69.9 (37.8)	75.9 (35.8)	72.2 (44.3)	52.4 (42.4)	68.4 (37.6)	0.590
Health Perceptions	55.3 (28.8)	62.9 (27.1)	63.9 (6.8)	57.1 (26.9)	42.1 (32.6)	0.101
Respiratory Symptoms	74.8 (23.5)	77.8 (19.8)	72.2 (13.6)	85.7 (17.8)	66.7 (29.4)	0.164
					
**Leicester Cough Questionnaire**						
Total	15.5 (4.4)	15.9 (4.4)	17.3 (2.2)	16.1 (3.8)	14.2 (5.1)	0.348
Physical	4.98 (1.4)	5.24 (1.2)	5.6 (0.8)	5.1 (1.3)	4.5 (1.6)	0.238
Psychological	5.23 (1.7)	5.4 (1.7)	5.8 (0.8)	5.4 (1.5)	4.7 (1.9)	0.426
Social	5.36 (1.6)	5.5 (1.5)	5.9 (0.9)	5.6 (1.3)	4.9 (1.8)	0.348

Abbreviations: 6MWT, 6-min walking test; BMI, body mass index; FEV_1_, forced expiratory volume in 1 s; FVC, forced vital capacity; FUP, follow-up; Kg, kilogram; m, metre; min: minutes; mMRC, modified Medical Research Council scale; MVPA, moderate-to-vigorous physical activity. Data are presented as n (%), mean ± SD or median (P_25_–P_75_). ^a^ *p* < 0.05 for comparison between ‘active + not sedentary’ vs. ‘inactive + sedentary’. ^b^ *p* < 0.05 for comparison between ‘inactive + sedentary’ vs. ‘active + sedentary’.^c^ *p* < 0.05 for comparison between ‘inactive + not sedentary’ vs. ‘inactive + sedentary’.

**Table 3:** Significant risk factors for inactivity and sedentary behaviour in the logistic regression analyses.

Variable	Univariate ^a^			Multivariable ^b^		
	OR	95% CI	*p* Value	OR	95% CI	*p* Value
Age (+1 year)	1.003	0.96 to 1.05	0.906	-	-	-
6-min walking distance (+1 m)	0.998	0.99 to 1.00	0.518	-	-	-
FEV_1_ (+1% predicted)	1.005	0.97 to 1.04	0.796	-	-	-
Number of hospitalisations during FUP (+1 unit)	1.200	0.80 to 1.793	0.089	-	-	-
Number of exacerbations during FUP (+1 unit)	1.540	1.23 to 2.65	0.045	2.192	1.12 to 4.28	0.021

Abbreviations: CI, confidence interval; FEV_1_, forced expiratory volume in 1 s; FUP, follow-up; OR, odds ratio. The OR represents the odds that the change of group to ‘inactive + sedentary’ will occur with exposure to the explanatory variable, against the odds of the outcome occurring in the absence of that exposure. The *p*-value is based on the null hypothesis that all ORs relating to an explanatory variable equal unity (i.e., no effect). ^a^ The variables analysed in the univariate analysis were age, 6-min walking distance, FEV_1_% predicted, number of hospitalisations during the follow-up and number of exacerbations during the follow-up.^b^ Hosmer–Lemeshow goodness-of-fit test, *p* = 0.76.
